# Supplementary material for: nanotatoR: a tool for enhanced annotation of genomic structural variants
Source: BMC Genomics. 2021 Jan 6;22:10. doi: 10.1186/s12864-020-07182-w (PMC7789800; doi:10.1186/s12864-020-07182-w)
Supplement: Supplementary file 1 — Additional file 1: Figure S1. Deletion variant on chromosome 4 identified in sample NA24385: Cartoon of the chromosome 4 region deleted in the NA24385 genome (a) and screenshot of the matching UCSC genome browser output (b). Breakpoints coordinates are shown as calculated by SVcaller in (a) and after including a method-average breakpoint error of +/− 3 kb [69,369,091 and 69,493,860] in (b). This variant deletes the entirety of coding gene UGT2B17, as well as the 3 pseudogenes UGT2B29P, AC147055.2 (or RP11-1267H10.2) and AC147055.4 (or RP11-1267H10.1), and ~ 63% of pseudogene AC147055.3. c) nanotatoR snapshot: The nanotatoR output indicates the overlap genes, the strand from which they are transcribed, and the percentage of the gene length overlapping with the deletion. It also displays the frequency (expressed as percentage) of the variant in the internal and BNDB databases, and the overlapping gene term with database info (osteoporosis found in the OMIM database). [file 12864_2020_7182_MOESM1_ESM.pdf]

a) **NA24385 Genome**

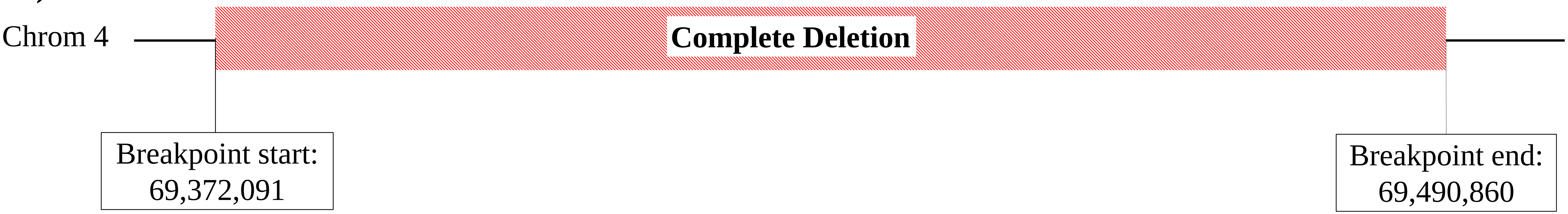

b) **UCSC Genome Browser**

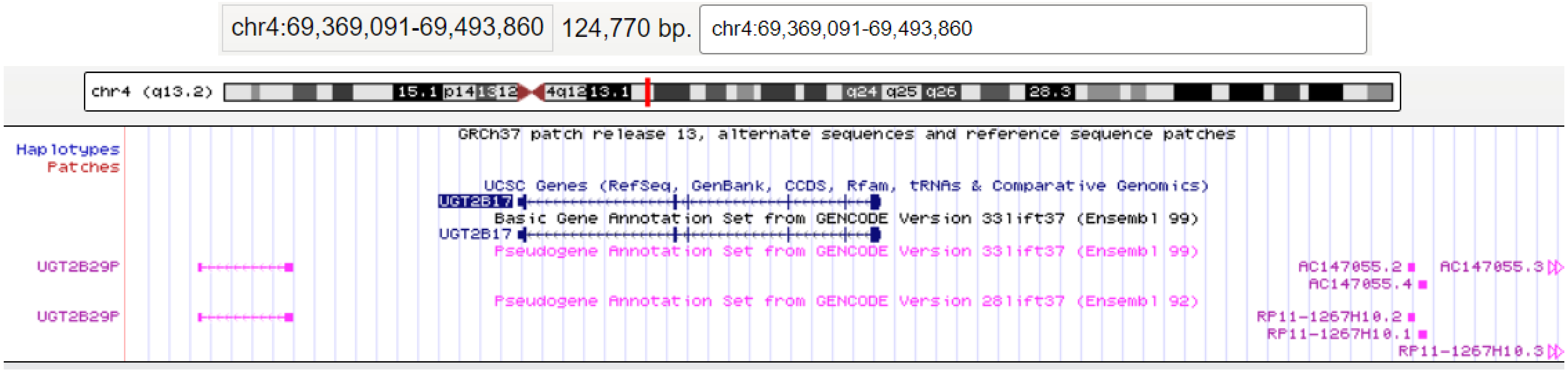

c) ***nanotatoR* Snapshot**

SV Type	Overlap Genes (strand;% overlap)	Internal Frequency	BNDB Frequency	Overlap Genes Terms
deletion	UGT2B29P(-:100) UGT2B17(-:100); AC147055.2(+:100) AC147055.4(-:100) AC147055.3(-;63.11)	30	9.188	UGT2B17(osteoporosis_OMIM)
